# The PrEP Pharmacy Reach Study: Protocol for the Creation of Maps to Visualize the Impact of Expanding Access to HIV Prevention Services Through Pharmacies

**DOI:** 10.2196/75077

**Published:** 2025-10-27

**Authors:** Kristin R V Harrington, Chante Hamilton, Daniel I Alohan, Alexis Hudson, Henry N Young, Natalie D Crawford

**Affiliations:** 1 Department of Epidemiology, Rollins School of Public Health, Emory University, 1518 Clifton Rd NE, Atlanta, GA, 30322, United States, 1 (404) 727-3956; 2 Department of Behavioral, Social and Health Education Sciences, Rollins School of Public Health, Emory University, Atlanta, GA, United States; 3 Department of Clinical and Administrative Pharmacy, College of Pharmacy, University of Georgia, Athens, GA, United States

**Keywords:** pre-exposure prophylaxis, HIV prevention access, pharmacies, geospatial, spatial analysis

## Abstract

**Background:**

Despite the proven efficacy of preexposure prophylaxis (PrEP) in reducing the risk of HIV transmission, uptake remains suboptimal among populations with limited access and availability to PrEP-prescribing locations, particularly in the Southern United States. The accessibility of pharmacies positions them as a promising resource for expanding PrEP delivery and access and supporting uptake and adherence through HIV prevention programs to reduce geographic disparities.

**Objective:**

This study outlines a geospatial protocol to assess disparities in national PrEP availability. The aim of this study is to develop a protocol to identify, map, and describe the potential impact of expanding PrEP access and HIV prevention services to pharmacies as alternative PrEP delivery sites across the United States. We propose a reproducible mapping and analytic framework to visualize gaps and inform implementation strategies at the state and local levels.

**Methods:**

We create local and state maps to help visualize the impact of expanding access to HIV prevention services through pharmacies. We obtain data from three main data sources: (1) pharmacy locations from the National Council for Prescription Drug Programs (NCPDP), (2) PrEP-prescribing facility locations from the CDC’s National Prevention Information Network (NPIN), and (3) HIV case data per 100,000 persons from AIDSVu. We geocode pharmacies and PrEP-prescribing locations with Google application programming interfaces (APIs) via the *ggmap* package in R software. Maps are created by overlaying several different layers of general maps and aggregated data including base maps, choropleth maps, dot density maps. To quantitatively examine the potential impact of expanding HIV prevention services, we calculate a PrEP facility-to-need ratio (PfnR) as the total number of facilities (PrEP-prescribing or pharmacies) divided by the number of HIV cases per 100,000 persons. Fold-change estimates are computed to quantify the increase in service reach if pharmacies were incorporated into PrEP delivery.

**Results:**

Overall, maps depicted far greater availability of local pharmacies compared to PrEP-prescribing facilities. When considered in the context of HIV cases per 100,000 persons, pharmacies were more prevalent than PrEP-prescribing facilities in areas with higher HIV caseloads. Mean PfnRs for pharmacies ranged from 0.04 (Mississippi) to 1.3 (Alaska), while PrEP-prescribing facility PfnRs were as low as 0.0004 (Puerto Rico). Estimated fold-change increases ranged from 6.4 in Idaho to 120.3 in Puerto Rico, with the greatest increases in the Southern and Midwestern United States.

**Conclusions:**

This protocol provides a scalable and reproducible framework to assess PrEP service distribution and to identify areas which may benefit most from pharmacy integration. Our findings suggest that incorporating pharmacies into PrEP-delivery could substantially reduce geographic access barriers, especially in underserved regions. This work has critical implications for state and national policies focused on avenues to increase PrEP access and uptake and subsequently reduce HIV transmission in their regions.

## Introduction

There are over 1.1 million people living with HIV in the United States, with almost 40,000 new cases diagnosed in 2022 [1]. Preexposure prophylaxis, or PrEP, significantly reduces the risk of HIV transmission, with an effectiveness of over 99% when taken consistently [2-4]. Inequities in PrEP uptake span across populations, risk groups and geographic regions. The overall rate of PrEP use in 2021 was 129 per 100,000 persons with the lowest rate of PrEP use among Latino populations and similar rates of use among Black and White populations [5]. People who inject drugs have a significantly increased risk of HIV transmission and consistently have some of the lowest rates of PrEP use compared to other key populations [6]. Further, although most men who have sex with men (MSM) are aware of PrEP, large discrepancies remain in reported PrEP use. For example, despite a greater need, about 15% fewer Black MSM report PrEP use compared to white MSM [7]. Although the Southern United States comprises over half of new HIV diagnoses and almost half of all HIV cases [1], PrEP use is relatively stagnant in this region, reflecting structural, socioeconomic, and health care access barriers leading to difficulties in access and availability of PrEP [6].

The Centers for Disease Control and Prevention, National Institutes of Health, and other public health organizations have recently emphasized the role of pharmacies in achieving the goals of the Ending the HIV Epidemic Initiative (EHE) [8,9]. EHE aims to reduce the number of new HIV infections in the United States by 90% by 2030, with a focus on priority jurisdictions with >50% of new HIV diagnoses [10]. Community pharmacies provide convenient and accessible care for the majority of Americans, with approximately 89% of the population living within 5 miles of one [11-13]. Their extended operating hours, including evenings, weekends, and holidays make them an ideal option for individuals who cannot visit clinics during standard hours [14]. This accessibility positions pharmacies as a vital resource for expanding PrEP access and supporting adherence, advancing public health efforts nationwide but especially in communities with fewer healthcare facilities [15,16]. Pharmacists have demonstrated their capability in supporting the provision of PrEP services from initiation to ongoing management [15,17,18]. Further, previous research highlights the potential of pharmacies as accessible sites for delivering HIV-related services, supported by a body of systematic literature reviews that examine the integration of HIV prevention resources, such as PrEP into pharmacy practice [19-22].

Inequities in access and availability of services within public health often display geographic patterns. given local differences in epidemiology, policies, and resources. Spatial mapping of disease patterns has the ability to identify high-risk regions and inform evidence-based public health interventions [23,24]. The use of maps in policy and decision-making offers substantial benefits, by enabling the identification of underserved regions, guiding the strategic allocation of resources and uncovering patterns and disparities that may not be readily apparent in other data representations [11,25].

Geospatial analyses have been instrumental in identifying inequities in the availability of HIV prevention services in areas with high HIV burden. Previous work has highlighted the limited geographic availability of PrEP clinics, particularly in the Southern United States where HIV prevalence is high, revealing significant gaps in service coverage for regions with substantial unmet need [26]. Other studies have demonstrated the utility of AIDSVu, a publicly available database and interactive mapping tool, in visualizing geographic inequities in HIV prevention and care, providing a useful tool for identifying areas where interventions are needed most [25]. Building on this foundation, recent work has demonstrated the potential of pharmacies to bridge these gaps by mapping their locations alongside PrEP prescribing sites in underserved communities [27].

This study builds on previous research by expanding mapping of pharmacies offering HIV prevention services, specifically PrEP, across the United States. By combining geographic data with key indicators such as HIV prevalence and socioeconomic factors, this study assesses the potential reach of pharmacies in addressing PrEP. This approach identifies geographic gaps in PrEP access, offering valuable information to inform the development of targeted strategies to address these disparities. We aim to identify, map, and describe the potential impact of expanding PrEP access and HIV prevention services to pharmacies across the United States.

The findings from this study have the potential to guide local and state-level policy initiatives aimed at enhancing PrEP accessibility through pharmacies. Additionally, this research may serve as a model for similar efforts in other areas of health care delivery, demonstrating how geographic data and health indicators can be used to optimize resource allocation. Ultimately, this work has broader implications for improving health equity by addressing inequities in HIV prevention and ensuring that at-risk populations have access to necessary healthcare services.

## Methods

### Overview

This paper outlines a protocol for the creation of maps to help visualize the impact of expanding access to HIV prevention services through community pharmacies. These maps allow for the examination of geographic distributions of current PrEP-prescribing locations compared with pharmacy locations. We further describe the creation of PrEP facility-to-need ratios to quantify the potential reach of pharmacies to expand PrEP access. Similar implementation of this protocol has been described previously [27].

### Data Sources

We used three main data sources for the creation of our maps. Pharmacy locations for pharmacies across the United States were obtained from the National Council for Prescription Drug Programs (NCPDP) [28]. This dataset included pharmacy street address, contact information, provider type, accessibility services, and levels of delivery services provided. PrEP-prescribing locations were obtained from the National Prevention Information Network (NPIN) [29]. This platform, managed by the Centers for Disease Control and Prevention (CDC), provided aggregated street addresses and latitude and longitude coordinates for PrEP-prescribing locations. PrEP-prescribing locations include any health care facility in which a licensed provider can prescribe PrEP, which may be a community health center, primary care clinic, or a specialized clinic. These facilities can provide general and specialized care services depending on their capacity and level of specialization of providers. Data on HIV cases per 100,000 persons were collected at the state, county, and zip code levels from AIDSVu for states, counties, and cities [30]. AIDSVu is a publicly available database and interactive mapping tool that integrates data from the CDC and local health departments [31]. Website users may explore the HIV epidemic using multiple outcome measures at different spatial scales. Datasets are freely available for download for different outcomes at various levels of spatial granularity.

### Geocoding

To geocode pharmacies and PrEP-prescribing locations, we used Google application programming interfaces (APIs) via the *ggmap* package in R software (version 4.4.3; R Foundation for Statistical Computing) [32]. This application converts street addresses to latitude and longitude coordinates that can then be plotted on maps. State, county, and zip code boundary files were gathered from the US Census Bureau using the R package *tigris* (version 1.5) [33]. Street addresses for all locations were geocoded to latitude and longitude coordinates. Quality checks were implemented at this stage, including plotting points and confirming coordinates were within correct state and county boundaries. For locations outside of expected boundaries, locations were manually geocoded using provided address.

### Map Creation

**Figure 1.**
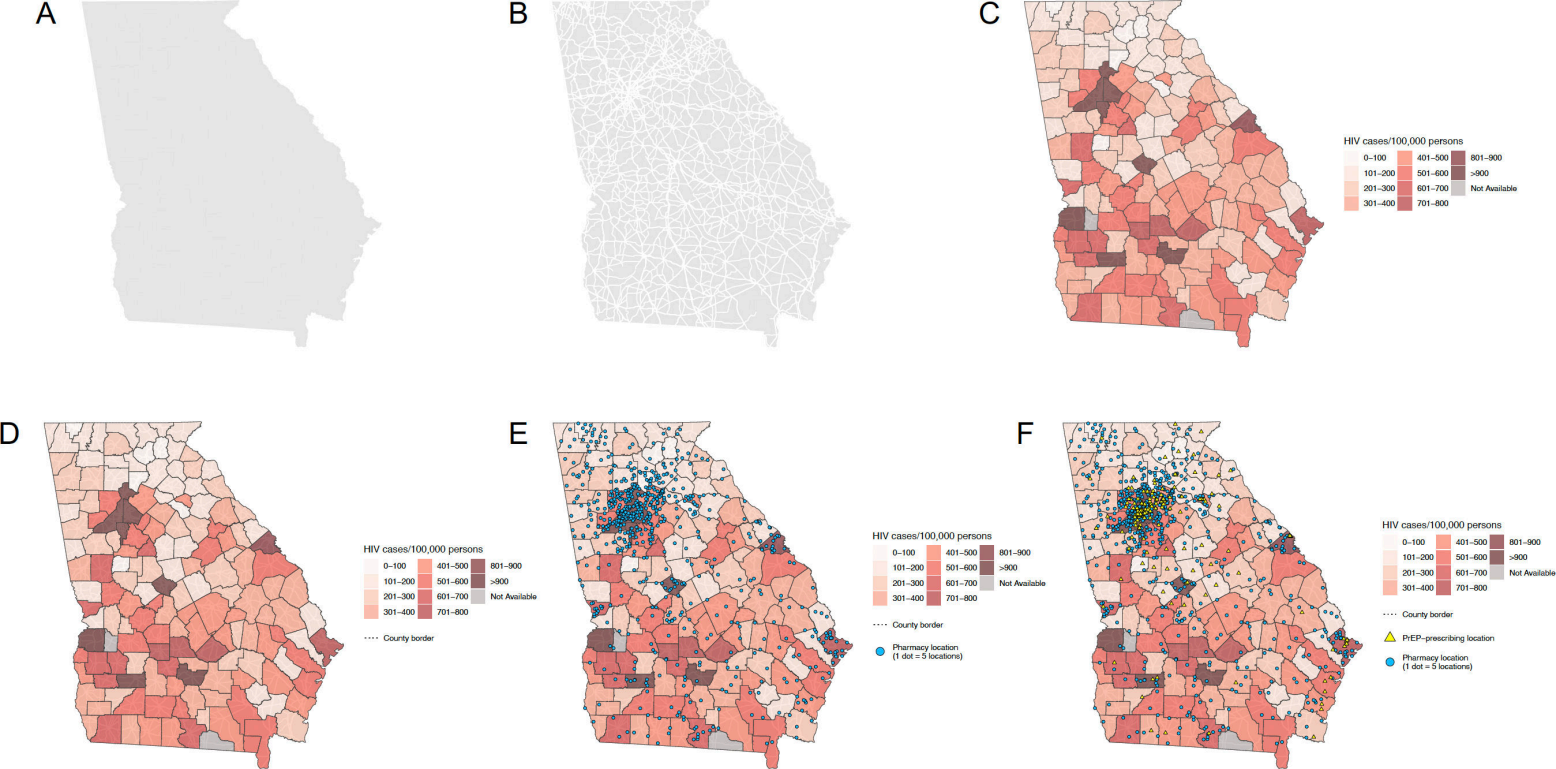
Sequential process of the creation of a state map.

**Figure 2.**
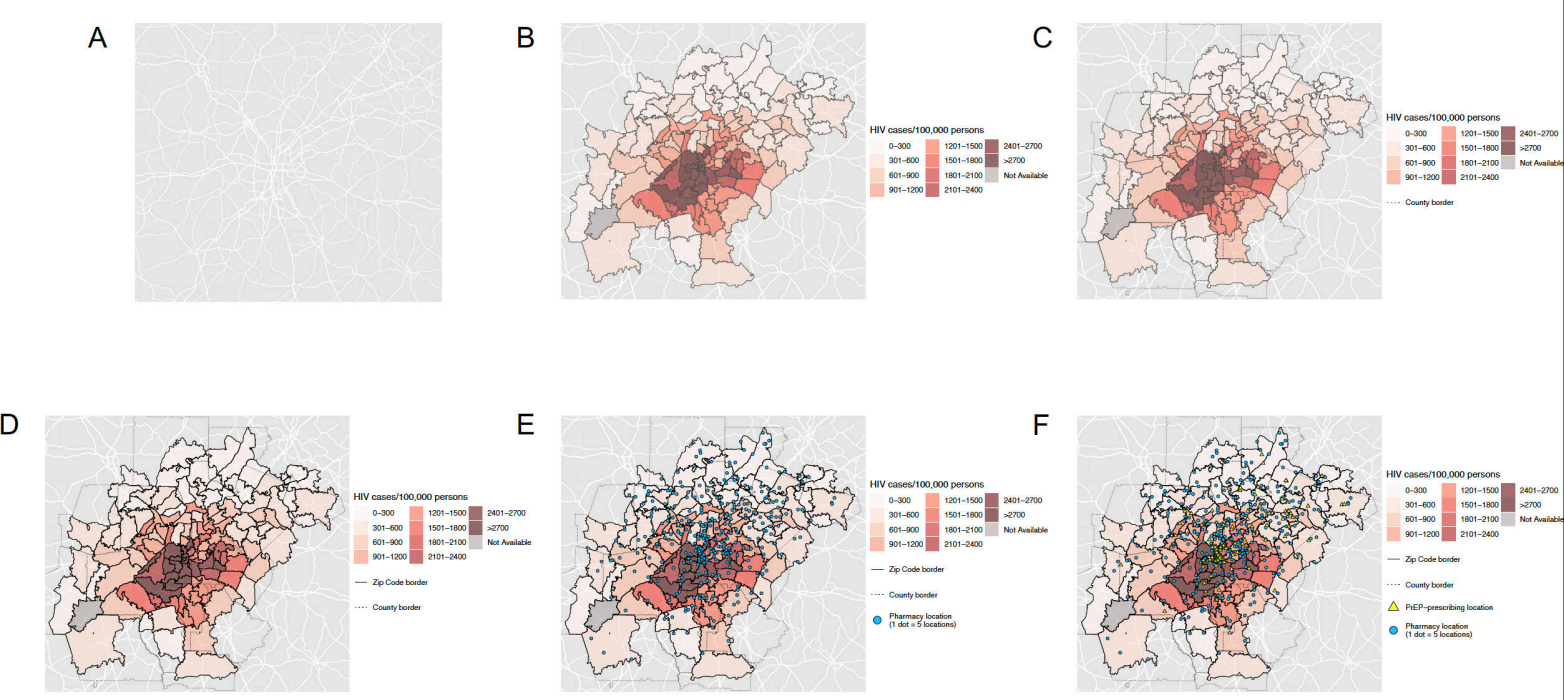
Sequential process of the creation of a city map.

**Figure 3.**
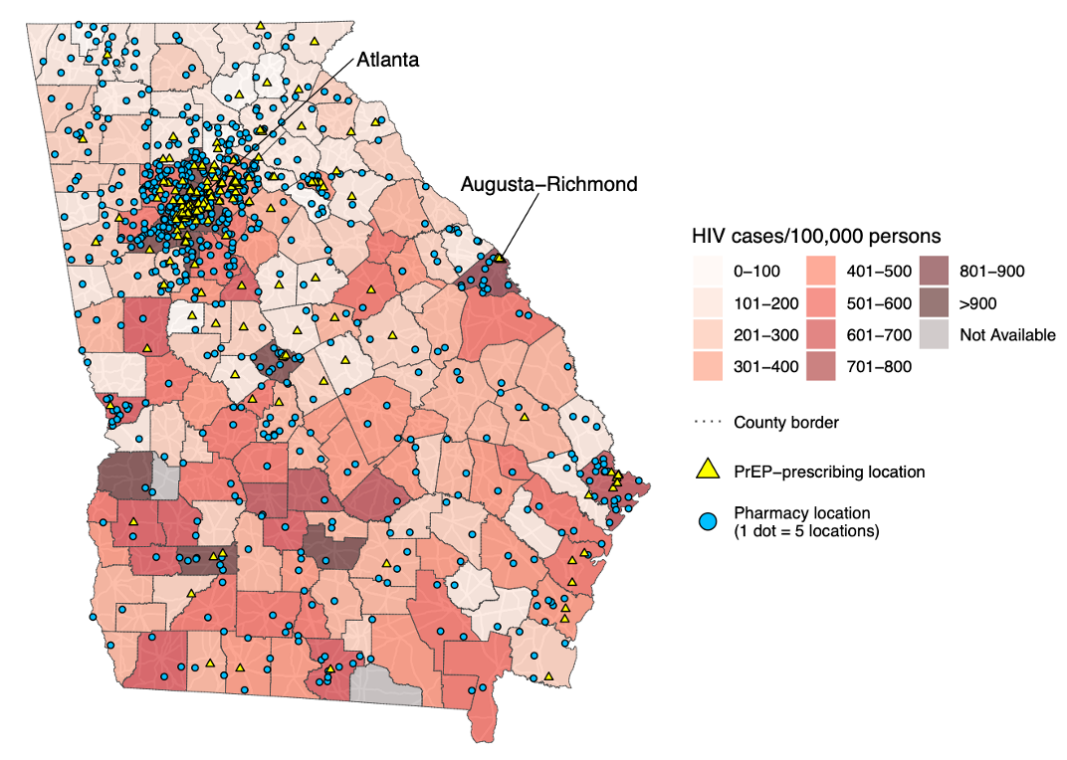
Completed state map of Georgia. PrEP: .

Maps were created by overlaying several different layers of general maps and aggregated data. Base maps including landmass areas and roads were obtained by using functions from the *sf* package in R [34,35]. Sequential steps in the creation of state and city maps are displayed in Figures1  2, respectively. First, landmass areas were plotted and overlaid with major roadways (Figures1A, B  2A). Next, a choropleth map was overlaid displaying the number of HIV cases per 100,000 persons (Figures1C  2B). Choropleth maps of HIV cases per 100,000 persons were indexed to the state, county, and zip code levels for either state or city maps depending on the available level of granularity of data from AIDSVu. To better distinguish spatial boundaries, county borders were then layered on top for both states and cities, followed by additional boundaries for zip code borders for cities (Figures1D, 2C  D). The next layer was a dot density plot for pharmacies, where one point corresponded to five pharmacy locations to better visualize locations given the generally large number of existing pharmacies (Figures1E  2E). This was subsequently followed by a point layer depicting the geocoded PrEP-prescribing locations, where each point represented a specific location (Figures1F  2F). Last, city labels were applied for further spatial context (Figures3  4).

**Figure 4.**
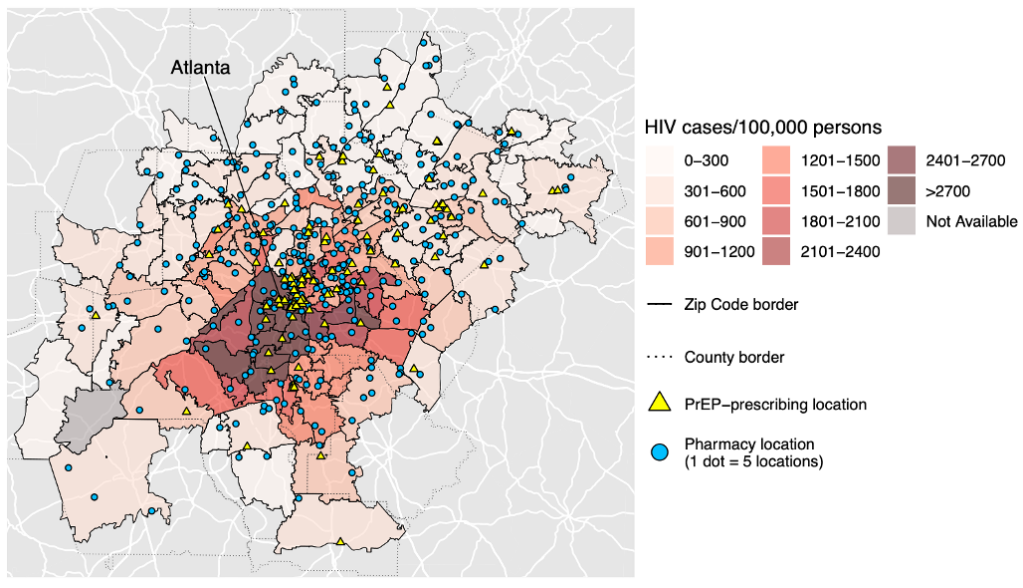
Completed city map of Atlanta. PrEP

### Statistical Analysis

To quantitatively examine the potential impact of expanding HIV prevention services, we calculated a PrEP facility-to-need ratio (PfnR) as the total number of facilities (PrEP-prescribing or pharmacies) divided by the number of HIV cases per 100,000 persons. Lower PfnRs indicate lower capacity to meet the needs of the HIV risk population. Additionally, we calculated the fold-change difference between PfnR estimates between PrEP-prescribing facilities and pharmacies within the same spatial area. Higher values indicate greater potential impact of expansion of HIV prevention services to pharmacies.

### Ethical Considerations

This study was conducted using publicly available and deidentified datasets. No individual-level protected health information was used, and no human participants were directly involved. Thus, institutional review board approval was not required.

## Results

Maps were created for 50 states, 54 cities, and one US territory. In general, maps depicted far greater accessibility to local pharmacies compared to PrEP-prescribing facilities. When taken in the context of HIV cases per 100,000 persons, pharmacies were more prevalent than PrEP-prescribing facilities in areas with higher HIV caseloads. These trends persisted at state, county, and zip code levels across the majority of states. Some regions had more aptly placed PrEP-prescribing locations across their areas including areas with higher HIV caseloads, while there were some states that had more rural regions with a paucity of both pharmacies and PrEP-prescribing facilities.

Mean PfnRs for pharmacy locations at the state level ranged from 0.04 in Mississippi to 1.3 in Alaska, with mean PfnRs for PrEP-prescribing facilities ranging from 0.0004 in Puerto Rico to 0.19 in Alaska. Estimated fold-increase for expansion of HIV prevention services to pharmacies was the lowest in Idaho (6.4) and highest in Puerto Rico (120.3). Overall, fold-increases were greatest for Midwestern and Southern states, and lowest for Western and Northeastern states.

## Discussion

This study presents a scalable and reproducible geospatial framework to identify and visualize disparities in PrEP access with a specific focus on evaluating the impact of incorporating pharmacies as a potential solution to improve geographic inequities. We found that pharmacies are significantly more prevalent than PrEP-prescribing facilities in almost all locations evaluated. The greatest impact of expanding HIV prevention services was observed in the Southern and Midwestern regions of the United States where HIV burden is disproportionately high. The calculated PfnRs and associated fold-change estimates highlight the potential for pharmacies to serve as key locations for expanding HIV prevention services.

Our work builds on prior studies demonstrating that PrEP availability often fails to align with population-level need [36], as well as recent literature emphasizing the logistical limitations of clinic-based PrEP delivery models [37,38]. States can address these limitations by leveraging the existing extensive network of community pharmacies to provide more equitable PrEP access [39]. Importantly, the PfnR used in this study serves as a novel indicator of PrEP delivery capacity relative to local HIV burden.

The ideal pharmacy-based PrEP delivery model would include both prescribing and ongoing management in collaboration with a clinician, spanning baseline eligibility assessments, lab testing, medication administration, adherence counseling, and follow-up monitoring [40]. However, expansion of HIV prevention services to pharmacies across the United States requires navigating state-level regulatory heterogeneity [41]. For states in which pharmacists lack the authority or reimbursement mechanisms to provide PrEP, operationalizing the model described here may be more challenging. Future work could incorporate state-specific policy data to evaluate the readiness of implementation. Additionally, future studies may evaluate the differences in impact of expanding HIV prevention services to all available pharmacies versus a subset optimized to either location or availability of resources.

To our knowledge, this was the first study to spatially examine the distribution of pharmacies and PrEP-prescribing facilities within the context of HIV prevalence across the United States. This work helps identify the areas of greatest need for PrEP availability and HIV prevention services. Further, the methodology used in this study has the potential to be applied to a number of other health conditions, spanning infectious diseases and non-communicable diseases. Other demographic indicators may be substituted in for disease prevalence if examining risk factors.

We acknowledge that this work is subject to a number of limitations. First, maps were limited to the granularity and accuracy of the data provided. Thus, for some states (eg, Alaska), the number of HIV cases per 100,000 persons was unavailable at the county or zip code level, so one estimate was provided for the entire state. This limits the ability to develop interpretations at a level more granular than the state level. Further, these differences in spatial granularity across regions limit our ability to make comparisons across dissimilar spatial scales (ie, comparing a state PfnR to a county PfnR in a different state). Regarding accessibility of data, some states do not provide HIV case data for all counties, resulting in several counties and zip codes with estimates that are unavailable. This data may be withheld by states or censored by AIDSVu for many reasons including privacy concerns if only very few cases live in a spatial area, or uncertainty in counts leading to unstable estimates. Lastly, our estimates do not account for variation in state regulations around pharmacy-based PrEP delivery and thus, these differences may limit implementation feasibility in certain jurisdictions. Despite these limitations, this study protocol can provide guidance around resource allocation and inform more equitable and geographically tailored PrEP expansion strategies.

The findings from this study have important implications for both research and policy. By providing a data-driven framework to visualize PrEP access gaps and estimate the potential of pharmacy-based delivery, this protocol offers a practical tool for public health agencies, policymakers, and health systems to guide resource allocation. The contribution of this project will be a robust examination of pharmacy locations with respect to HIV caseload across the United States. By using NCPDP data, it is likely that we were able to capture all pharmacy locations in the country, resulting in more accurate estimates of the potential impact of expanding HIV prevention services to pharmacies. This work has critical implications for state and national policies focused on avenues to increase PrEP access and uptake and subsequently reduce HIV transmission in their regions.

## Abbreviations

API: application programming interfaces
CDC: Centers for Disease Control and Prevention
EHE: Ending the HIV Epidemic Initiative
MSM: who have sex with men
NCPDP: National Council for Prescription Drug Programs
NPIN: National Prevention Information Network
PfnR: PrEP facility-to-need ratio
PrEP: preexposure prophylaxis
